# Dental Management of a Young Child Affected by Galactosialidosis and a Gigantic Abdominal Growth

**DOI:** 10.1155/2018/2086157

**Published:** 2018-04-01

**Authors:** Yoselín Méndez-Salado, Paola De Ávila-Rojas, Amaury Pozos-Guillén, Raúl Márquez-Preciado, Miguel Ángel Noyola-Frías, Socorro Ruiz-Rodríguez, Arturo Garrocho-Rangel

**Affiliations:** Pediatric Dentistry Postgraduate Program, Faculty of Dentistry, San Luis Potosi University, San Luis Potosi, SLP, Mexico

## Abstract

Galactosialidosis (GS) is a rare form of lysosomal storage disease that involves a broad spectrum of skeletal and soft tissue abnormalities. We report here on a 4-year 7-month-old boy with mild mental retardation, exhibiting multiple caries cavities and associated infectious foci and macroglossia. A huge abdominal enlargement due to peritoneal ascites was evident. Behavioral management and patient positioning on the dental chair represented a true challenge. The patient was treated under general anesthesia. However, life-threatening postoperative complications occurred because of the impossibility of extubating the patient. A very careful preanesthetic assessment is crucial in children affected by general conditions associated with airway anomalies, such as GS.

## 1. Introduction

Lysosomal storage diseases (LSDs) are uncommon metabolic disorders produced by an accumulation of glycoconjugates (glycosaminoglycans, glycoproteins, or glycosphingolipids) within lysosomes, which affects normal tissue architecture in diverse areas of the human body. LSDs can be very rare individually; however, as a group, these anomalies comprise around 70 pathologic conditions with an incidence of 1 : 5000 live births [1, 2]. *Galactosialidosis* (GS) is an uncommon lysosomal storage condition, which belongs to the glycoproteinosis subgroup of LSDs, inherited as an autosomal recessive trait [3]. The prevalence of the disease is, to our knowledge, unknown. It is caused by genetic mutations in the CTSA gene, which causes a deficiency or reduced activity of the protective protein cathepsin A (PCCA) [4, 5]; PCCA forms a complex with other two glycosidases: beta-galactosidase (beta-GAL) and neuraminidase 1 (NEU1). Thus, loss of PPCA function results in a severe secondary deficiency of NEU1 and partial deficiency of beta-GAL [6]. As a consequence of this, there is poor formation of elastic fibers, which are essential components of the connective tissues that form the body's supportive framework [7]. The diagnosis of GS is therefore made by measuring the activity of PCCA and by confirming the secondary deficiency of beta-GAL and NEU1 [8].

Children with GS present with a broad spectrum of clinical manifestations. Based on the age of onset of the disease during childhood and disease severity, GS is classified into three subtypes [3]. The *early infantile* type initiates between 0 and 3 months of age. It is the most severe form of GS and is associated with premature mortality [9, 10]. This form includes fetal hydrous (or *hydrops fetalis*, a severe life-threatening edema in the fetus or newborn), abdominal hernias, ascites, coarse face, proteinuria, telangiectasia (abnormal dilation of the superficial capillaries, arterioles, or venules, typically localized immediately below the skin surface), skeletal dysplasia (dysostosis multiplex, stippled epiphyses, and osteoporosis), nephrotic syndrome, cardiac failure, neurological deficit, and ocular defects. The most affected patients die early, within the first year of life, due to renal and cardiac involvement. The second subtype, *late infantile*, begins during the first 2 years of life, slowly progressing into adulthood. It is characterized primarily by absent or mild neurological/cognitive disability and mental deterioration. This condition consists of dysostosis multiplex, especially of the spine, growth retardation-associated muscular atrophy, hepatosplenomegaly, cardiac involvement (thickening of the heart valves), macroglossia, and hearing loss [1, 3, 9]. Additionally, there is a third subtype of GS, the *juvenile/adult* type. It represents the most common form of the disorder (approximately 60%), and it is more prevalent in the Japanese population. This subtype is characterized by the presence of myoclonus (a sudden and involuntary jerking of a muscle or group of muscles), ataxia, angiokeratoma (a skin condition manifested by clusters of dilated blood vessels, thickened skin, and warty growths), mental retardation, and long survival [8].

We describe the specific clinical body and oral characteristics of a 4-year 7-month-old male patient affected by early infantile GS and mild mental retardation, who presented an enormous abdominal enlargement. Due to this anatomical condition, there were diverse difficulties regarding the oral approach and body positioning of the patient on the dental chair.

## 2. Case Report

A 4-year 7-month-old boy and his parents were referred to the Pediatric Dentistry Postgraduate Program Clinic in June 2016, requesting dental treatment due to multiple dental caries cavities, local infectious processes, and associated pain. Two years previously, the patient had been diagnosed with early infantile GS, confirmed on the analysis of the beta-GAL both in peripheral blood leucocytes and in cultured skin fibroblasts (sequencing of the CTSA gene was not carried out). Previously, the child was insufficiently treated by a pediatric dentist, due to the child's very poor level of cooperation. Only the upper right anterior segment was treated: a pulpectomy procedure on the lateral incisor and extraction of the root remnant of the central incisor. However, the patient did not continue the treatment.

Medical and dental history revealed that when the child was 1 year of age, his parents noticed the existence of a mild soft outpouching swelling in his lower abdomen, which progressively increased in size. The patient was evaluated at a local public hospital, and the condition was diagnosed as peritoneal ascites, together with three abdominal hernias, due to enlarged liver and spleen.

**Figure 1:**
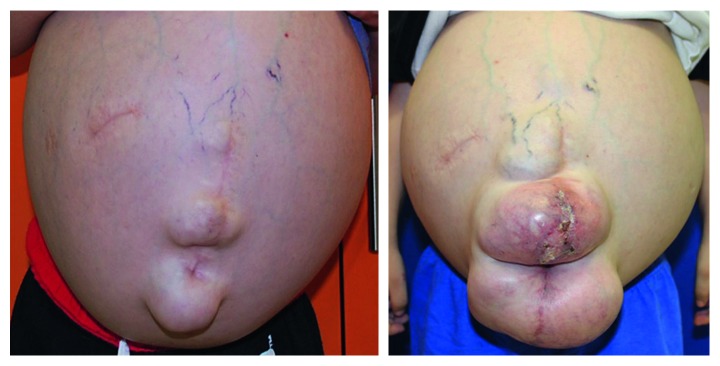
Abdominal enlargement: at the initial visit (June 2016) and at the last control appointment (November 2017).

At the moment of the patient's first dental visit, the presence was evident of a huge abdominal growth due to ascites (Figure 1). According to the treating medical team, this anomaly was unable to be surgically repaired. Because of the significant swelling, the patient had difficulty in maintaining a straightened body posture, and he could not be adequately positioned on the dental chair. In addition, the patient manifested mild mental retardation, language delay, severe bilateral hypoacusia, hepatic damage, and bilateral hydrocele (swelling in the scrotum).

**Figure 2:**
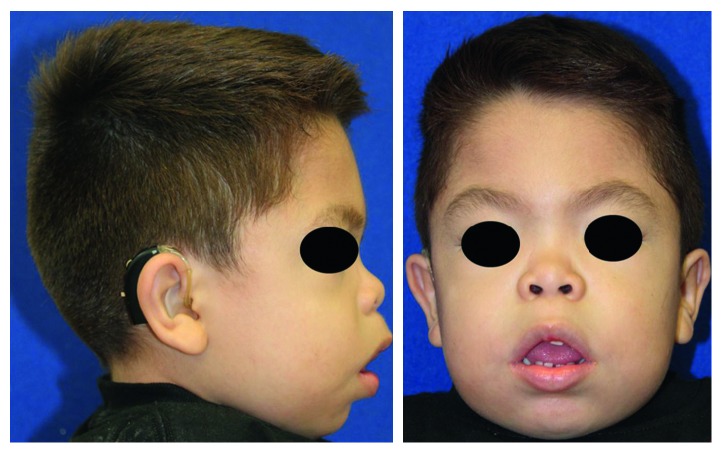
Extraoral views. Note the audition appliances.

**Figure 3:**
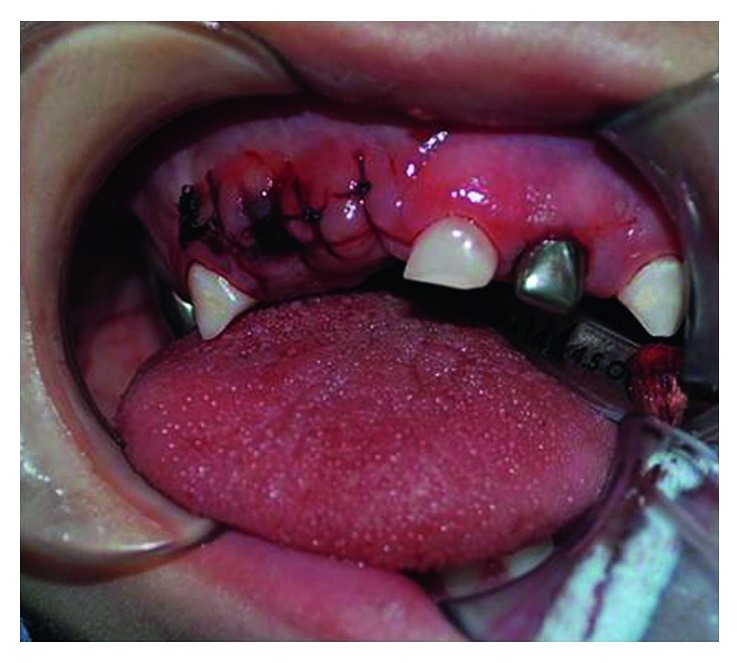
View of the macroglossia.

The patient's head exhibited a squared form, coarse face, and short neck. The facial profile was markedly convex with an increased lower third, retrusive chin, protruding maxilla, closed nasolabial angle, and manifested lip incompetence (mouth permanently open) (Figure 2). Intraorally, the examination showed both arches with interdental spacing, carious cavities in all primary molars, a root remnant of the upper left lateral incisor with related abscess fistula and gingival swelling, and macroglossia associated with an evident anterior open bite (Figure 3). Oral hygiene was very poor, and halitosis was significant.

The programmed treatment plan consisted of the placement of composite restorations, pulpotomies and preformed metallic crowns, and extraction of the root remnant. Due to the greatly reduced level of cooperation exhibited by the patient (rated as Frankl's scale level I, definitely negative), it was not possible to obtain X-rays. The patient was very fearful, with clear evidence of treatment refusal, forceful crying, and extreme negativism. Therefore, it was decided to start the treatment with an oral examination, dental prophylaxis, topical fluoride-varnish applications, and the teaching of tooth brushing. Traditional behavioral management techniques, such as conditioning, desensitization, “tell-show-do,” and positive reinforcement, were persistently employed. On the other hand, the patient was unable to maintain a supine or horizontal position on the dental chair due to pain caused by the abdominal hernia. Thus, the patient was approached when he was in a 90-degree seated position, with the aid of his mother. However, all these efforts were unsuccessful. Then, it was decided to treat the patient under general anesthesia, in agreement with the parents, who signed a special informed consent document.

The patient was managed according to the American Academy of Pediatric Dentistry (AAPD) guidelines on sedation and general anesthesia. First, the child was sent to the pediatric anesthesiologist for a physical examination and a presurgical health and risk evaluation; respiratory, cardiovascular, and gastrointestinal systems were exhaustively assessed, and blood and urine laboratory tests were indicated; only coagulation times appeared slightly increased. The patient was classified as American Society of Anesthesiologists (ASA) physical status classification III, with lower pulmonary capacity, limited open aperture, macroglossia, and challenging airway access due to decreased diameter. The parents were instructed, through printed guidance, regarding their child's eating and drinking on the day prior to the intervention.

The surgical intervention was carried out in August 2016 at the university hospital. After placing routine monitors, according to the American Society of Anesthesiologists standards, general anesthesia was induced via facemask with inhaled fentanyl, lidocaine, propofol, rocuronium bromide, and sevoflurane. Supplemental local anesthesia was also provided at the site of the root-remnant extraction. The extraction site was fully sutured with fine absorbable 6-0 Dexon in order to prevent a potential hemorrhagic episode. The whole surgical procedure lasted approximately 2 hours and ensued without complications. However, the extubation procedure was not possible due to respiratory restriction, and the patient was subsequently transferred to the pediatric intensive care unit (PICU). After 4 days under pharmacological management (dexamethasone, metamizole, ephedrine, clindamycin, and midazolam), together with assisted mechanical ventilation, the extubation could finally be performed. The patient was remitted to the pediatric area, where he was maintained with oxygen nebulization; the case proceeded uneventfully thereafter. He was discharged from the hospital 2 days later.

The patient was evaluated at our clinic 15 days after the intervention conducted under general anesthesia. Restorations were found to be in place adequately, and the cicatrization process at the extraction site was uneventful. Then, an individualized oral preventive program was initiated, including dental hygiene practice with a fluoridated paste (1,450 ppm), topical fluoride varnish, MI Paste Plus® applications, and diet counseling. Since then, the patient has been reviewed closely, every month; at each of the visits, the previously mentioned behavior modification techniques were applied in depth. The last control appointment took place in mid-November 2017, during which an excellent oral condition was observed. Currently, the patient is considered a poor candidate for treatment with orthodontic appliances, particularly for treating his anterior open bite. In the meanwhile, the eruption process and occlusal development will be continuously assessed.

## 3. Discussion

The American Academy of Pediatric Dentistry (AAPD) has recognized that “providing both primary and comprehensive preventive and therapeutic oral health care to individuals with Special Health Care Needs (SHCNs) is an integral part of the specialty of pediatric dentistry,” and SHCNs are defined as “any physical, developmental, mental, sensory, behavioral, cognitive, or emotional impairment or limiting condition that requires medical management, healthcare intervention, and/or use of specialized services or programs” [11].

In this report, we present a rare case of a mentally challenged pediatric patient with early infantile GS dentally treated under general anesthesia, who exhibited a critical postoperative adverse effect associated with a compromised airway. Due to the rarity of this condition, such clinical case reports are lacking in the dental literature. To the best of our knowledge, this is the first case describing a child with early infantile GS reported in the pediatric dentistry literature.

GS is a condition of metabolism classified as a lysosomal storage disease associated with soft tissue aberrations; some of these present in the orofacial complex, for instance, macroglossia and adenoidal/tonsillar hypertrophy. Also frequent is a decreased airway diameter [1, 10]. This condition can represent a real challenge for diagnosis and clinical care in pediatric dentistry, for example, diverse significant implications during the dental management with general anesthesia, as occurred in the present case. Despite the potential difficulty being detected at the preanesthetic evaluation and in spite of the anticipated preoperative medical measures being taken, the very problematic extubation could not be avoided. This information confirms that general anesthesia cannot be considered by pediatric dentists as an “easy” solution to manage “difficult” children, nor is there a completely safe procedure [12]. However, in the case of our patient, his significant anatomical condition and lack of cooperation were sufficiently justified selection indicators for carrying out the procedure, despite the high risk involved.

## 4. Conclusions

Pediatric dentists should always pay special attention to and be aware of the potential risks of disorders that can produce life-threatening conditions. Also, a very careful preanesthetic assessment is crucial in children affected by general conditions or syndromes associated with airway anomalies, such as GS.
